# Lable-free aptamer portable colorimetric smartphone for gliadin detection in food

**DOI:** 10.3389/fbioe.2024.1338408

**Published:** 2024-02-19

**Authors:** Yadi Qin, Sicheng Zhang, Jie Qian, Fanxing Meng, Jun Yao, Minwei Zhang

**Affiliations:** ^1^ School of Pharmacy, Xinjiang Medical University, Urumqi, China; ^2^ College life Science and Technology, Xinjiang University, Urumqi, China; ^3^ Key Laboratory of Active Components and Drug Release Technology of Natural Medicines in Xinjiang, Xinjiang Medical University, Urumqi, China

**Keywords:** gliadin, aptamer, smartphone, colorimetric sensor, RGB values

## Abstract

For individuals with celiac disease (CD), the current clinical therapy option available is a lifelong gluten-free diet. Therefore, it is essential to swiftly and efficiently detect gluten in foods. A colorimetric sensor has been developed, which operates by regulating the aggregation and dispersion state of AuNPs induced by high concentration NaCl through the specific binding of gliadin and aptamer, thereby achieving rapid detection of gliadin in flour. It is found that the sensor exhibits good linearity in the concentration range of 0.67–10 μM and the LOD (3σ/S) is 12 nM. And it can accurately distinguish various types of free-gliadin samples, with a spiked recovery rate of 85%–122.3%. To make the detection process more convenient, the colorimetric results of the biosensor were translated into RGB color-gamut parameters by a smartphone color-picking program for further analysis. Gliadin can still be accurately quantified with the established smartphone platform, and a correlation coefficient of 0.988 was found. The proposed portable smartphone aptamer colorimetric sensing device has achieved satisfactory results in the rapid detection of gliadin in food.

## 1 Introduction

Celiac disease (CD) is an immune deficiency disorder that affects the small intestine and various other organs. The global prevalence of CD is approximately 1%, with significant variations observed between different countries and regions (De Re et al., 2017; Lebwohl et al., 2018). The disease’s etiology may be associated with genetic factors, autoimmune levels, and environmental factors (Kuja-Halkola et al., 2016), but the consumption of gluten-containing foods is considered the primary cause of CD. Hitherto, the only clinical therapy option available is a lifetime gluten-free diet (Glissen Brown and Singh, 2018; Li et al., 2023). Gluten is a protein mixture occur in grains (especially wheat, barley, and rye), mainly composed of gliadin and glutenin (Tye-Din et al., 2018; Tanner et al., 2019; Bahmani et al., 2024), among which gliadin is the culprit causing celiac disease (Menezes et al., 2023). Therefore, many countries have established guidelines for gluten-free and extremely low gluten content foods and set a minimum detection threshold for gluten at 20 mg/kg (Commission, 2014). The existing rapid detection methods for gluten are mainly based on enzyme-linked immunosorbent assays (ELISA) (Lacorn et al., 2019; García-García et al., 2020; Sajic et al., 2020), which have the characteristics of rapid and high sensitivity. However, the ELISA is not fully compatible with the organic solvent extract of gluten. It is prone to inducing denaturation, resulting in abnormal or false-positive test results. In addition, proteomic detection methods based on polymerase chain reaction (PCR) and traditional instrumental analysis methods based on mass spectrometry (MS) have also been used for the detection of gluten (Colgrave et al., 2015; Fiedler et al., 2018; Garrido-Maestu et al., 2018; García-García et al., 2019). These methods generally require longer detection times and expensive laboratory equipment.

In recent years, aptamers have attracted widespread attention as chemical antibodies. Small oligonucleotide sequences or short peptides called aptamers are produced using the Systematic Evolution of Ligands by Exponential Enrichment (SELEX) method (Cai et al., 2018; Buglak et al., 2020). These aptamers have a high affinity and excellent selectivity for binding with corresponding ligands (Mieczkowski et al., 2021; Kadam et al., 2022), which can include viruses, proteins, entire cells, etc. The domains of chemical biology and biomedicine now have a new, effective, and quick recognition research tool attributable to their development. And it has shown great application prospects in the rapid detection of various substances (Lei et al., 2022; Chen et al., 2023; Dong et al., 2023). In the past few years, high specific aptamers apt-1 (Amaya-González et al., 2014) and apt-2 (Svigelj et al., 2020) of gliadin have been reported and used to construct fluorescent and electrochemical aptamer sensors (Amaya-González et al., 2015; López-López et al., 2017; Pla et al., 2021). Although these aptasensors exhibit high sensitivity and selectivity, there was still room for improvement in the simplicity required for on-site testing of gluten using these methods.

**FIGURE 1:**
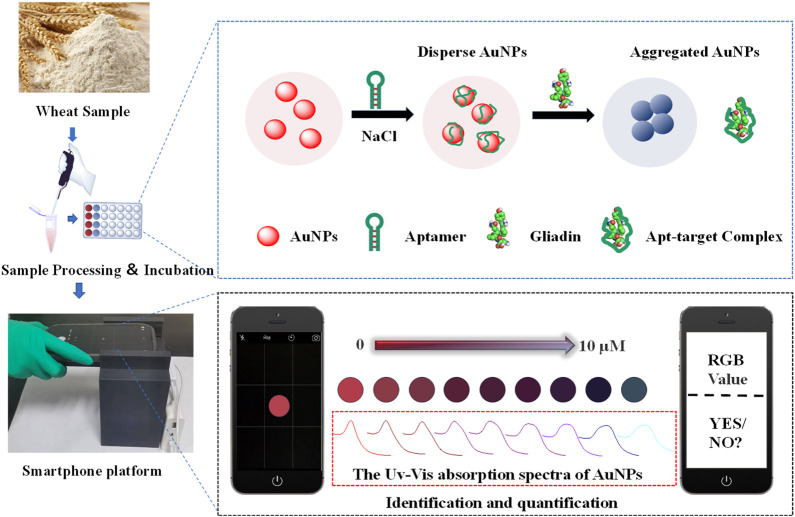
Schematic representation for a visual colorimetric smartphone platform for rapid identification of gliadin in food.

With the development of computing and multitasking performance, sensors based on intelligent sensing platforms have emerged as a promising solution for converting laboratory analysis processes into on-site, real-time monitoring (Yang et al., 2017; Mao et al., 2019). Among them, optical aptasensors based on smartphones have the characteristics of portability and high sensitivity. The principle is mainly to establish an RGB color model through a smartphone color-picking program. The RGB color model captures pixel values at specified positions through established techniques, and defines the amount of red, green, and blue light in the XYZ 3D coordinate model through red, blue, and green values. The combination of these color components defines a single color. The target substance can be quickly and conveniently analyzed by colorimetric analysis using this RBG chromaticity space color parameter (Fay and Wu, 2024; Li et al., 2024). In this work, a colorimetric sensing device has been constructed. The principle is shown in Figure 1, AuNPs were used as colorimetric signal reporters, and aptamers are recognition components. As the gliadin concentration gradually increases, the sensing signal can be converted into color gamut parameters through the colorimetric analysis system of smartphones. It is easy to detect gliadin in various grain alcohol extracts. Meanwhile, it was investigated at how well different wheat gliadin aptamers performed in terms of detection when used to build colorimetric sensors. The proposed adaptive smartphone device has ability to accurately quantify gliadin. It can meet the minimum requirements of the EU for gliadin testing. The entire detection process is rapid and simple, without the need for any additional instruments. Due to the 3D cassette design, this method is more suitable for use outside the laboratory and has stronger adaptability to the environment compared to most current detection methods. It has great potential for application in on-site detection of gliadin.

## 2 Materials and methods

### 2.1 Materials and reagents

Shanghai Macklin Biochemical Co. Ltd (Shanghai, China) contributed the gliadin from wheat (CAS: 9,007–90–3), HAuCl_4_·3H_2_O, dimethyl silicone oil, Tris-HCl buffer (1 M, pH 5.0/6.8/7.4/8.0/8.8), and sodium chloride. The supplier of trisodium citrate dihydrate was Tianjin Yongsheng Fine Chemicals Factory (China). Anhydrous ethanol was obtained from Tianjin Damao Chemical Reagent Company (China). Aptamer probe (the sequence is as follows: Apt-1: CCA GTC TCC CGT TTA CCG CGC CTA CAC ATG TCT GAA TGCC, Apt-2: CTA CAC ATG TCT GAA TGCC) was purchased from Sangon Biotech (Shanghai, China), Flour, sorghum powder, millet, milk powder, oats, and coconut powder were all purchased from the local market.

### 2.2 Apparatus

The Mettler AB135-S electronic analytical balance was used for sample weighing (METTLER TOLEDO Ltd, Switzerland). UV spectrum was scanned at 400–800 nm using a UV-2700 spectrophotometer (SHIMADZU, Japan). DF-101S collector type constant temperature heating magnetic stirrer (Changzhou Putian Instrument Manufacturing Co., Ltd, China) was used for the preparation of AuNPs. The form and distribution of AuNPs were ascertained with a JEM-1230 transmission electron microscope (JEOL Co., Japan). Shanghai Liantai 3D Lite600 was used for 3D printing of smartphone cartridges (China).

### 2.3 Details of the combining mechanism’s simulation

The three-dimensional structure of Gliadins was retrieved (CAS: 9,007–90–3) as docking ligands from the PubChem database, The 3D structure of the aptamer was predicted through the RNAComposer online simulation service platform. And it was optimized for geometry and energy through the ChinmeraX 1.4 software. Subsequently, the Lamarckian genetic algorithm of the AutoDock 4.2 software was used for multiple docking calculations, and the combination of free energy was used as the basis for evaluating the final docking form. Pymol 2.1 was used for analyzing the docking data.

To further investigate the molecular mechanism of gliadins binding to aptamers, MD modeling of aptamer target complexes was performed using the Amber 2020 program. The receptor used AMBER99SB-ILDN force field parameters, while ligands used gaff universal force field parameters. Based on the docking results, the TIP3P dominating water model was chosen, and sodium or chloride ions were utilized to neutralize the system charge. The target-aptamer complex was simulated for 400 ns using four steps: energy minimization, heating, balance, and production dynamics simulation. Trajectory data every 100 ps was saved and the trjconv module was used to perform correlation analysis. By applying the MMPBSA method, the binding free energy of both ligands and receptors was further calculated.

### 2.4 The preparation of gold-nanoparticles

The preparation of AuNPs was carried out using the method of reducing HAuCl_4_ with sodium citrate. Overall, all glassware was soaked in 18% dilute nitric acid for 24 h and then washed with ultrapure water to remove the interference of foreign ions. 100 mL of 1 mM HAuCl_4_ solution was heated and continuously stirred until boiling in an oil bath (180°C). After rapidly adding 10 mL of a 38.8 mM sodium citrate solution, the wine-red AuNPs solution was produced by heating and stirring constantly for 10 min. The solution was cooled to room temperature and stored in a brown bottle for experimental operation, and the molar concentration of AuNPs will be calculated by Lambert Beer’s law.

### 2.5 Lable free aptamer-AuNPs sensor for glutelin detection

300 μL AuNPs and 200 μL 40 nM aptamer solution was thoroughly mixed for 10 min into Tris-buffer (5 mM, pH 7.4), the mixed solution was then given 200 μL of various gliadin concentrations, and it was incubated for 10 min. Finally, 200 μL 40 mM NaCl solution was injected and vortexed for 30 s. The final volume is 900 μL. The absorbance ratios A_650_/A_520_ and color changes of AuNPs mixed solutions at different concentrations were record. Additionally, RGB values will be obtained for further analysis using the smartphone and the Color-Picker program.

### 2.6 Samples treatment

To investigate the recognition ability of aptasensor for gluten in food samples, 0.5 g of flour, oat, millet, milk powder, sorghum powder, and coconut powder were weighed and added to 10 mL ethanol-H_2_O solution (8:2, v: v). The mixture was extracted by sonicating at 70°C for 90 min, then centrifuged at 3,000 rpm for 15 min. Finally, the supernatant was collected, dried with nitrogen gas at 40°C, and redissolved in 10 mL ethanol-H_2_O solution (1:9, v: v) to obtain the final solution to be tested. The extraction process of other proteins in flour mainly refers to previous research methods (Daly et al., 2022). The difference in color and ΔR of each food sample extraction solution was recorded in the aptasensor (ΔR = R-R_0_, R is the absorbance ratio of each food sample extract in the aptasensor, and R_0_ is the absorbance ratio of the blank solution).

### 2.7 Fabrication of portable colorimetric-smartphone device

A cassette (85 mm * 85 mm * 135 mm) was designed by Computer-Aided Design (CAD) program and 3D printing was carried out. The device design includes an LED cold light strip, a pull-out storage slot, and a self-made color comparison 16-hole plate (hole diameter 8 mm). Every smartphone scan can be positioned 10 cm vertically above the sample owing to the pull-out structure.

## 3 Results and discussion

### 3.1 Characterization and principle of the gliadin-aptamer-AuNPs colorimetric system

**FIGURE 2:**
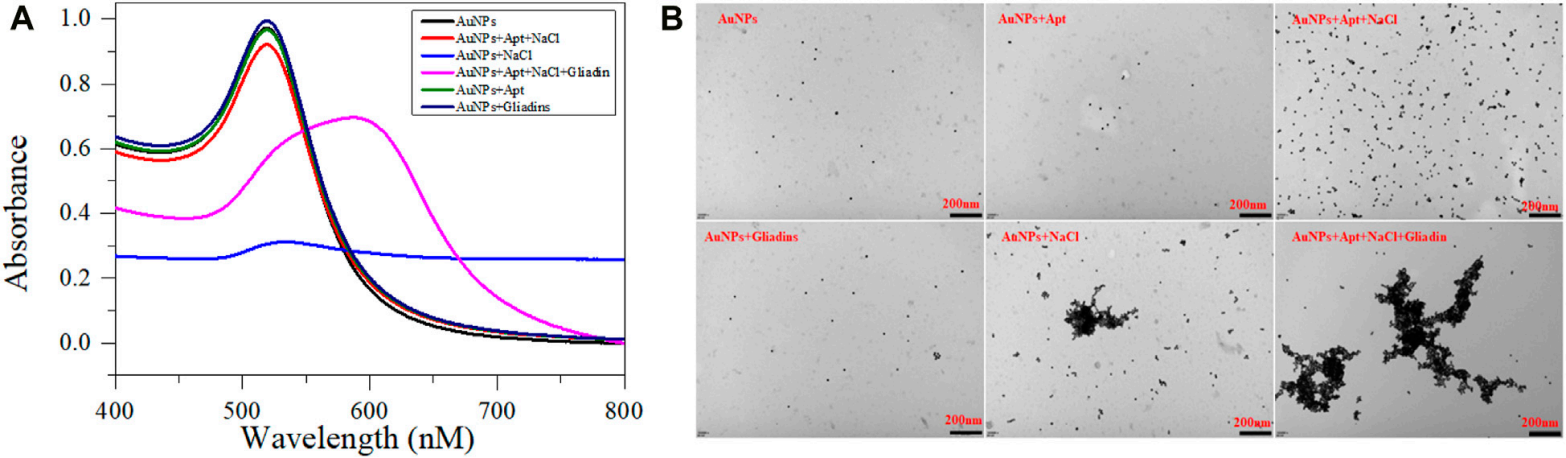
UV absorption spectra **(A)** and TEM images **(B)** of prepared AuNPs mixed with different solutions. The AuNPs is 300 μL, NaCl is 200 μL (37 mM), Aptamer is 200 μL (40 nM), Gliadin is 200 μL (10 μM) respectively.

As a metal nanomaterial, AuNPs with a high absorption coefficient, high extinction coefficient and highly SPR. It provides excellent sensitivity for constructing colorimetric sensors based on aptamer (Gong et al., 2017). The synthesized AuNPs have an average diameter of roughly 13 nm (Supplementary Figure S1) and a concentration of 3.53 nM (Qin et al., 2022). The pureS AuNPs solution can exhibit a stable dispersion state and appear red. After mixing high concentration NaCl with AuNPs solution, the static equilibrium of the solution was disrupted, and the plasmon band shift between gold nanoparticles caused rapid changes in the color and aggregation state (Figure 2). Meanwhile, AuNPs have a large specific surface area and can adsorb aptamers onto their surface through electrostatic adsorption (Priyadarshni et al., 2018). Therefore, after adding the aptamers, NaCl cannot cause a change in the state of AuNPs. Finally, when Gliadin is introduced into the mixed solution, Gliadin will preferentially combine with the aptamer to form a stable complex, so that AuNPs will leak out and be induced to aggregate by NaCl. The change in the aggregation and dispersion states of AuNPs can also cause ordered changes in the UV absorption spectrum of the entire system. It is worth noting that neither the individual aptamer nor gliadins will cause a change in the state of AuNPs. Therefore, AuNPs can be used to construct aptasensors.

### 3.2 Optimization of sensing conditions

**FIGURE 3:**
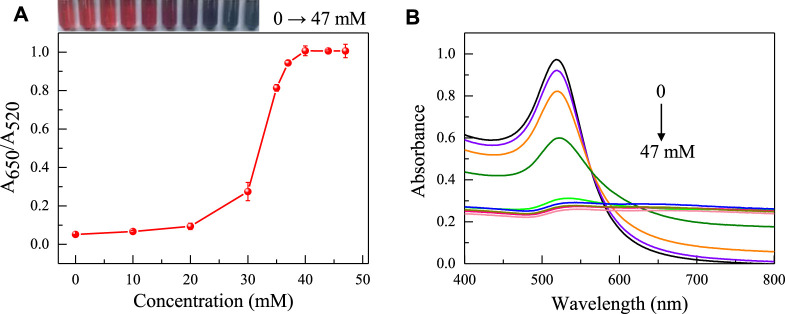
The influence of NaCl concentration on the absorbance ratio **(A)** and the UV absorption spectra **(B)** of AuNPs in the sensing system (The concentrations of NaCl is 0, 10, 20, 30, 33, 37, 40, 43, 47 mM respectively). The color changes of the sensing system are shown in the inset picture.

Due to various factors may interfere with the detection process of colorimetric sensors, it is crucial to optimize the sensing conditions to ensure the accuracy and sensitivity of the aptasensor. The priority optimization is the concentration of NaCl that causes AuNPs to aggregate. The aggregation degree of AuNPs was mainly evaluated by measuring the color and absorbance ratio (A_650_/A_520_) of the solution. As shown in Figure 3, as the concentration of NaCl gradually increases, the solution’s coloring shifts from red to purple to blue, and the A_650_/A_520_ also gradually increases. At a NaCl concentration of 37 mM, there were no significant changes in the color and absorbance ratio of the solution, indicating that AuNPs had fully aggregated. The concentration of 37 mM is the optimal concentration for adding NaCl.

**FIGURE 4:**
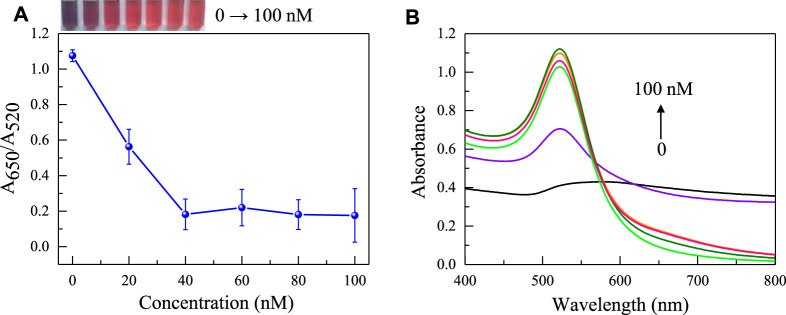
The effect of aptamer concentration on the absorbance ratio **(A)** and the UV absorption spectrum **(B)** of AuNPs in the sensing system (The concentrations of NaCl is 0, 20, 40, 60, 80, 100 nM respectively). The color changes of the sensing system are shown in the inset picture.

**FIGURE 5:**
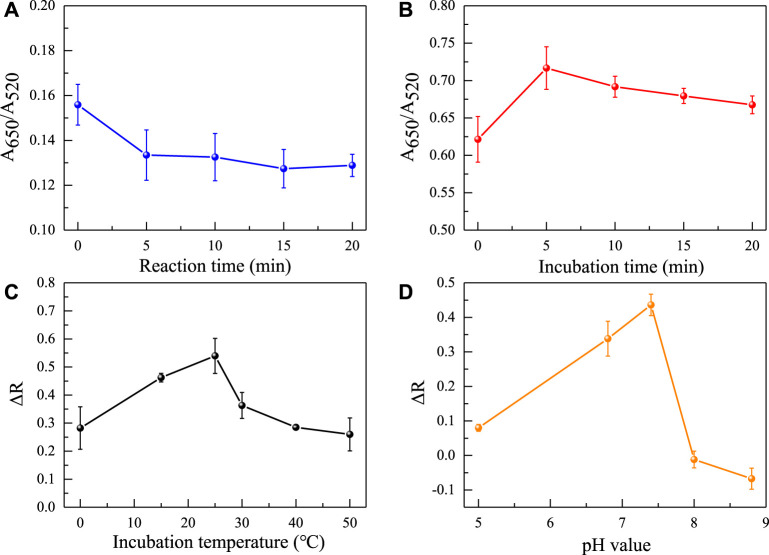
Effects of different reaction times **(A)**, incubation times **(B)**, reaction temperatures **(C)**, and pH values **(D)** on the absorbance ratio of the sensing system.

Afterwards, the concentration of the aptamer added as an anti NaCl aggregation agent in the sensing system was also evaluated. As the concentration of the aptamer gradually increases, it can better protect the uniform dispersion of AuNPs from the influence of NaCl solution. When the concentration of the aptamer is greater than 40 nM (Figure 4), the aptamer completely adheres to the surface of AuNPs, the solution changes from blue to red. Because it takes a reaction time for the aptamer to be adsorbed to AuNPs and incubation time for the aptamer to combine with gliadin, the influence of time factors on the sensing system was investigated. It was found that the time factors had no significant effect on the A_650_/A_520_ of the system after the solution was fully mixed. This also shows that the constructed aptamer sensor was suitable for on-site rapid detection of gliadin. However, for the best detection performance, the incubation time and reaction time were both set at 5 min. Finally, the pH value and reaction temperature of the sensing system were investigated. As shown in Figure 5, it was found that the aptasensor exhibited the best performance in a neutral tris (pH 7.4) buffer solution at 25 °C.

### 3.3 Evaluation of selectivity and performance of colorimetric sensor for detecting gliadin

**FIGURE 6:**
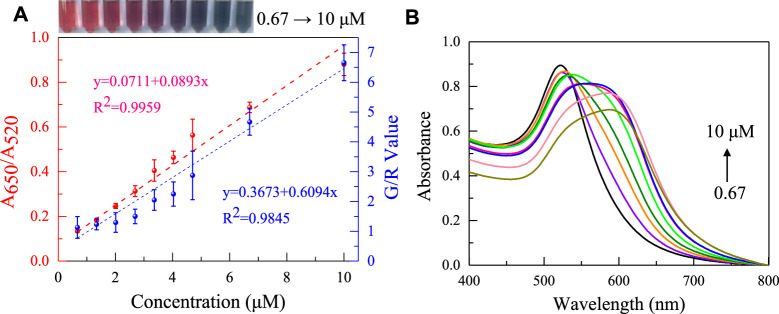
**(A)** The absorbance ratio of the apt-1 colorimetric sensor changes with the addition of concentrations of 0.67–10 μM gliadin (red dashed line), and the smartphone platform records the changes in G/R values with concentrations of 0.67–10 μM gliadin (blue dashed line). **(B)** Absorption Spectra of aptasensors with gliadin at different concentrations.

Under the optimized conditions, the analytical performance of the designed aptasensor for gliadin was further evaluated. Colorimetric sensors use two gliadin aptamers that have been reported. As shown in Figure 6 (red line) and Supplementary Figure S2, the A_650_/A_520_ of AuNPs increases steadily as the gliadin concentration increases, and the solution’s colors shift from red to purple to blue gray. The two aptamer sensors showed a good linear relationship (apt1: y = 0.0711 + 0.0893x R^2^ = 0.995/apt2: y = 0.0768 + 0.0819x R^2^ = 0.997) in the gliadin concentration range of 0.67–10 μM. However, the colorimetric sensor based on Apt1 shows higher sensitivity to gliadin, and the detection limit is 12 nM (3σ/S). Interestingly, Apt-2 (Kd = 148 nM, LOD is 13.08 nM) has a higher affinity for gliadin than Apt-1 (Kd = 186 nM) (Amaya-González et al., 2014; Svigelj et al., 2020), which contradicts the sensitivity exhibited by the two aptasensors to gliadin. It is due to the different adsorption abilities of AuNPs on two different length aptamer sequences. The sensitivity of AuNPs-aptamer based sensors is not only affected by the interaction between the aptamer and the target, but also by the interaction between AuNPs and the aptamer. To ensure optimal detection performance for gliadin, subsequent studies were based on the aptamer Apt-1. It is worth noting that the increase in the concentration of gliadin causes a significant color change, which provides a basis for building colorimetric smartphones.

**FIGURE 7:**
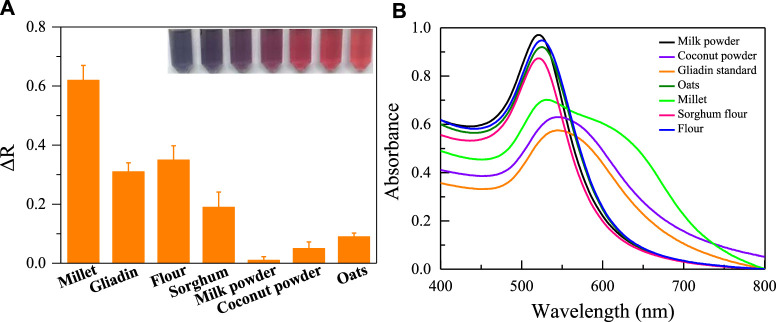
**(A)** The selectivity of the established colorimetric sensor for analysis of agricultural products with gliadin; **(B)** The UV absorption spectra corresponding to the tested agricultural products.

The key to the successful operation of the aptasensor was the specific binding of aptamer with gliadin. Therefore, the selectivity and reliability of the colorimetric sensor based on aptamer Apt-1 to detect gliadin were assessed using the ethanol extracts of six different agricultural products. As shown in Figure 7. For the alcohol extracts of flour, sorghum flour, and millet containing gliadin, the absorbance ratio of the sensor significantly increased, and the color of AuNPs changed from red to blue. For other agricultural products that do not contain gliadin (such as milk powder, oats, coconut powder), the absorbance ratio and color changes can be ignored. In addition, the interference of other proteins in the flour on the sensing system was also investigated. As shown in Supplementary Figure S3, Wheat Glutenin, Wheat globulin and Wheat albumin do not cause significant changes in the colorimetric system. Meanwhile, these proteins do not bind with bare AuNPs to cause significant changes in the absorbance and color of AuNPs. This indicates that aptamer sensor based on Apt-1 has excellent selectivity for recognizing gliadin.

To further test the suitability of the colorimetric sensor for detecting gliadin in food, 0.8, 2 and 10 μM gliadin standard were added to two types of gliadin free samples and flour samples, respectively. The samples were extracted with ethanol and analyzed using the established colorimetric sensor to calculate the recovery rate. Considering the high content of gliadin in flour, the flour sample was diluted to match the detection range of the aptasensor. The results are shown in Table 1. The spiked recovery rate is between 85%–122.3%, it is indicatd that the established aptasensor can be successfully applied to the quantification of gliadin residue levels in food and further demonstrates that the sample matrix will not affect the aptamer sensor.

**TABLE 1:** Analysis of results of gliadin in food samples.

Sample	Added (μL)	Found (μL)	Recovery (%)	RSD (%)
Milk powder	0.8	0.83	103.7	5.13
	5	4.89	97.8	2.51
	10	10.82	108.2	3.87
Coconut powder	0.8	0.82	102.5	6.41
	5	5.12	102.4	4.23
	10	11.25	112.5	4.45
Flour	0.8	0.68	85	7.42
	5	5.89	117.8	6.15
	10	12.23	122.3	8.79

### 3.4 Study on the binding mechanism between aptamers and gliadin

**FIGURE 8:**
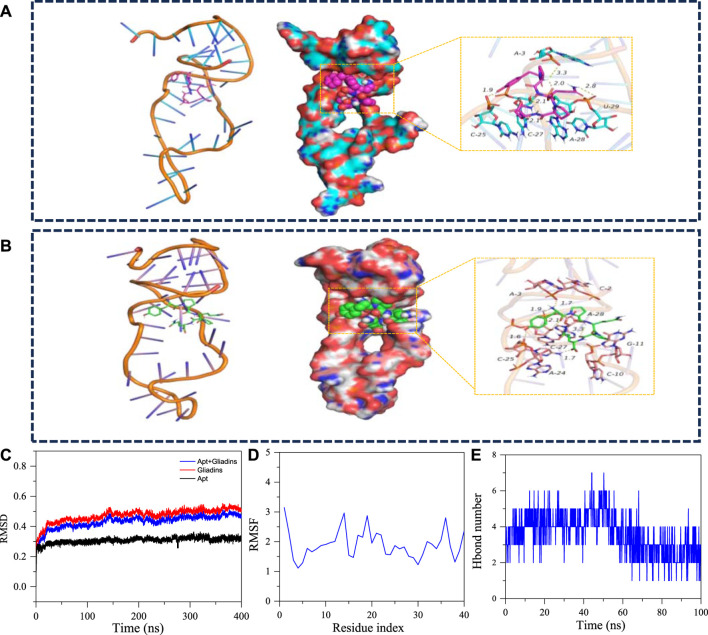
**(A)** The 3D structure and detailed binding mode of the apt-gliadin after molecule-docking. **(B)** The three-dimensional structure and detailed binding mode after MD. **(C)** The RMSD of the apt-1 after 400ns molecular dynamics. **(D)** The RMSD of the apt-1 after 400ns molecular dynamics. **(E)** The hydrogen bond number of Gliadins with apt-1.

To further investigate the binding mechanism between aptamers and gliadin. In this study, Macromolecular docking software was used to simulate the binding of aptamer Apt-1 to gliadin. As shown in Figure 8A, the sites involved in specific binding in the aptamer sequence include C-25, A-3, C-27, U-29, A-28 and other bases. Gliadin can form multiple hydrogen bonds with the A-28, U-29, A-3 bases. Hydrogen bonds have an average distance of 2.37 Å, and the length of hydrogen bonds is far less than 3.5 Å. It is indicating that the target had strong binding with the active site of the aptamer. To gain a more intuitive understanding of the binding between the aptamer and the target, the number of hydrogen bonds during the entire simulation process was detailed (Figure 8E). According to the hydrogen bond network diagram of receptors and targets, it can be easily found that gliadin can form two or more hydrogen bonds with aptamer bases, and these hydrogen bonds play a significant role in binding stable targets to receptors.

The binding free energy was a fundamental tool for analyzing changes in ligand binding modes by measuring the thermodynamic properties of ligands. A system’s stability is indicated by a negative value of binding free energy (Δ G binding energy), whereas instability is shown by a positive value. According to Supplementary Table S1, Van der Waals force has a high performance in stabilizing small molecules, followed by electrostatic interactions. This indicated that gliadin can be stably maintained in the pocket formed by the receptor site to form a stable van der Waals force with surrounding bases. In addition, due to the effective hydrogen bonding interaction between the target and the aptamer, electrostatic interactions also make an important contribution to stabilizing small molecules. In summary, aptamers have strong affinity for gliadin, which can promote them to form stable complexes and play an active role.

In addition, molecular dynamics simulations were used to further simulate the dynamic changes in the binding process between the aptamer and the target. As shown in Figure 8B, the binding site between the aptamer and the target had changed, and the bases that interact between them mainly included A-3, A-28, C-25, C-27, etc. Meanwhile, the conformation of the aptamer also changed, which led to a more stable conformation of the complex. In addition, the position of gliadin shifted to some extent, but it can still form stable hydrogen bond interactions with multiple bases (A-3, A-28, C-25, C-27). In the whole process of molecular dynamics simulation, gliadin always combined in the cavity formed by the aptamer, and there was no separation phenomenon. The total of all atomic position discrepancies between the current conformation and the original conformation at a specific time was known as the mean-square deviation, or RMSD. Throughout the whole simulation process, the monitoring system’s RMSD can give a comprehensive grasp of the complex’s structure and conformation, quantify the system’s stability, and ascertain the molecules’ degree of flexibility. It can be seen from the RMSD diagram that the average RMSD of the complexes is less than 3.8 Å, and the complexes reach dynamic equilibrium in a relatively short time, which indicates that the aptamer matches the target well and can form stable complexes. The base location with the largest fluctuation during the simulation process is represented by the peak, and the root mean square fluctuation can be used to quantify the conformational variations of each base on the physical chain. Greater base conformational variation and more flexible base residue mobility are associated with higher Root-Mean-Square Fluctuation (RMSF) values. According to the RMSF diagram, the overall base conformation changes were relatively small, which was the main reason for the minimum overall RMSD changes of the aptamer. In summary, aptamers have strong affinity for gliadin, which can promote them to form stable complexes and play an active role.

### 3.5 Construction of portable colorimetric smart phone for gliadin detection based on Aptamer-AuNPs

After the successful verification of the gliadin colorimetric sensor, a smart phone was designed to more quickly and visually detect gliadin (Supplementary Figure S4). The principle of this device is mainly to base the Android software (Ten thousand Color Extractor, version 5.2.0) to convert the colorimetric results of the aptamer sensor into color gamut parameters, and quickly analyze them by changing the ratio of green to red values (G/R values). However, conversion accuracy is greatly affected by environmental factors. As shown in Table 2; Supplementary Figure S5, the sensor solution was subjected to color analysis at different times. It was found that there is a significant difference in G/R values, which is caused by different light intensities at different time periods. In addition, due to the refraction of light by the liquid surface, different shooting angles from 45° to 90° can also cause serious interference in color-selection. Meanwhile, the device relies on a smartphone camera for color-picking, and the shooting focal length is also an important indicator. It was found that the phone-camera is basically unable to focus on the solution when the focal length is 3 cm. When the focal length is greater than 10 cm, the color-picking effect is the best. However, due to the influence of phone pixels, the color selection effect decreases when the focal length is greater than 20 cm.

**TABLE 2:** List of Changes in RGB Values Obtained from smartphone picking under different conditions.

Sample	Picking time (G/R value)			Camera angle (G/R value)			Focal length (G/R value)		
	am 0:00	am 8.00	pm 2:00	45°	60°	90°	3 cm	10 cm	20 cm
Blank sample	3.85 ± 0.03	1.85 ± 0.02	1.65 ± 0.03	2.74 ± 0.05	2.45 ± 0.01	1.57 ± 0.03	1.11 ± 0.02	1.45 ± 0.04	1.89 ± 0.04
Blank sample (cassette)	1.35 ± 0.02	1.33 ± 0.03	1.36 ± 0.01	-	-	1.36 ± 0.03	-	1.38 ± 0.02	-
Spiked sample	3.54 ± 0.04	4.61 ± 0.05	5.81 ± 0.04	3.89 ± 0.03	1.27 ± 0.02	5.57 ± 0.03	3.75 ± 0.04	6.21 ± 0.04	4.87 ± 0.03
Spiked sample (cassette)	6.01 ± 0.04	5.89 ± 0.03	5.92 ± 0.02	-	-	5.88 ± 0.05	-	6.11 ± 0.03	-

Spiked sample: gliadin with a concentration of 6 μM; mean and standard deviation results (n = 3).

To ensure the accuracy of the detection results, priority was given to ensuring the stability and consistency of the color-pick environment. Therefore, a cassette using 3D printing technology was designed and printed entirely with black polylactic acid (Supplementary Figure S4). The cassette is lined with black absorbent material (specially processed polyester polyurethane foam), which is a material that can block internal random reflections from visible light to infrared light (250–2000 nm) and the hemispherical reflectance is below 1%. It can effectively prevent stray light and internal reflection during the color-pick process. In addition, he vertical distance between the phone camera and the storage desk was set to 10 cm to ensure perfect focus during the color selection process.

Subsequently, a color-pick program running on a smartphone was used to convert the colorimetric results of the aptasensor into RGB values for quantitative detection of gluten. As shown in Figure 6A (blue line), The G/R value of the AuNPs solution rose as the gliadin content increased, and there was strong linearity in the gliadin concentration range of 0.67–10 μM. Different concentrations of standard samples were determined using absorbance ratio and smartphone methods, respectively, and it was found that the measurement results were basically consistent. And smartphone device analysis was more convenient and accurate compared to traditional naked-eye colorimetric methods. Meanwhile, the smartphone platform has has advantages and application prospects in the field of gliadin detection compared to existing detection methods (Table. 3).

**TABLE 3:** Comparable methods for determination of gliadin.

Method	Linear range	LOD	Recovery	Ref.
SERS	1 × 10−15-2 × 10-6 M	1.16 × 10−16 M	96.34%–104.44%	Fu et al. (2024)
Electrochemical Immunosensor	0.1–100 μg/mL	0.95 μg/mL	93.4%–103.3%	Zhan et al. (2023)
NIR spectroscopy	-	-	-	Wu et al. (2023)
Lossy Mode Resonance (LMR)	0.1–100 ppm	0.05ppm	-	Karamdoust et al. (2023)
Fluorescent nanosensor	0.36–2.20 μM	0.1 μg/mL	-	Benítez et al. (2023)
Colorimetric smartphone	0.67–10 μM	12 nM	85%–122.3%	this assay

## 4 Conclusion

In summary, a portable smartphone colorimetric device based on free-label apatmer-AuNPs has been constructed to quickly detect gluten in food. The smartphone detect platform can quickly analyze the colorimetric results of biosensors through a smartphone color-selection program, and has demonstrated good sensitivity, specificity, and convenience with gliadin. The detection performance of aptasonser meets the minimum requirements of EU for gluten detection in food. Meanwhile, due to the presence of 3D printing cartridges, the established smartphone sensing device can cope with various external laboratory environments, and the entire detection process is fast and does not require the use of other analytical instruments. It has great potential for application in gluten detection in food.

## Data Availability

The original contributions presented in the study are included in the article/Supplementary Material, further inquiries can be directed to the corresponding authors.
