# Supplementary material for: Lable-free aptamer portable colorimetric smartphone for gliadin detection in food
Source: Front Bioeng Biotechnol. 2024 Feb 19;12:1338408. doi: 10.3389/fbioe.2024.1338408 (PMC10910070; doi:10.3389/fbioe.2024.1338408)
Supplement: Supplementary file 1 [file DataSheet1.pdf]

Supporting Information for

Lable-Free Aptamer Portable Colorimetric Smartphone for Gliadin Detection in Food

Yadi Qin[1], Sicheng Zhang[1], Jie Qian[1], Fanxing Meng[2], Jun Yao[1,3*], Minwei Zhang[2 *]

[1] School of Pharmacy, Xinjiang Medical University,830017 Shangde North Road Urumqi, China.

[2] College life science & technology, Xinjiang University, 830046 Shengli Road Urumqi, China.

[3] The Project of Key Laboratory of Active Components and Drug Release Technology of Natural Medicines in Xinjiang, Xinjiang Medical University,830017 Shangde North Road Urumqi, China.

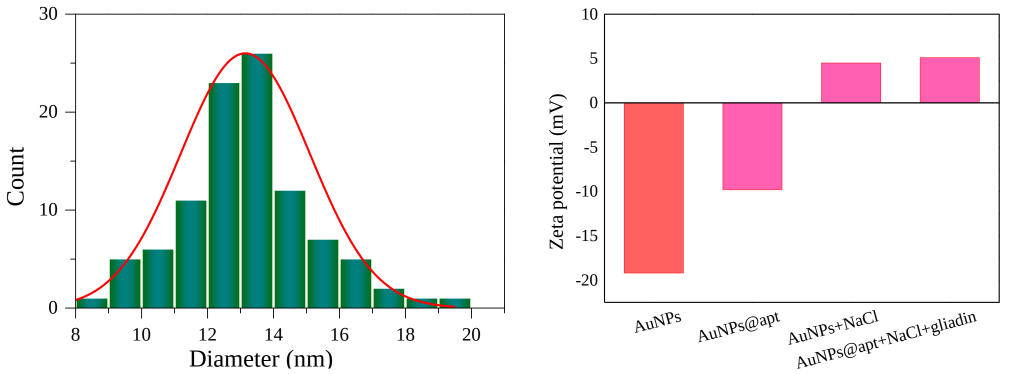

Figure S1. Statistical Diagram of Size Distribution of AuNPs(The average size of AuNPs is about 13nm), And the zeta potential diagrams of AuNPs in different solutions.

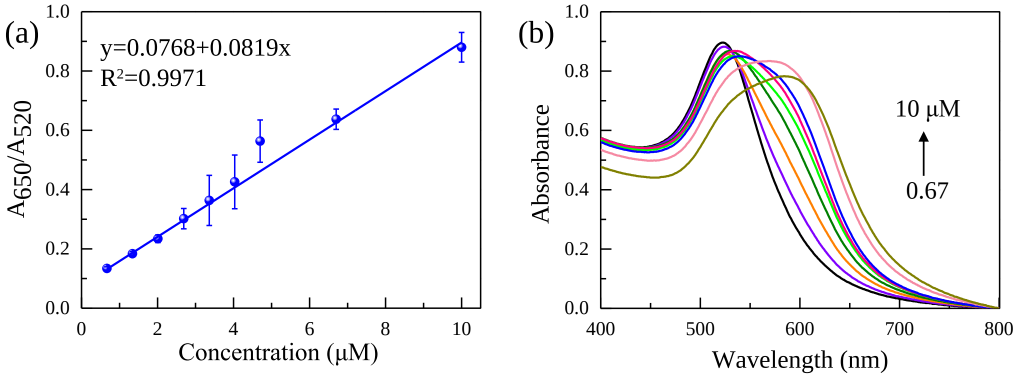

Figure S2. (a) The absorbance ratio of the apt-2 colorimetric sensor changes with the addition of concentrations of 0.67-10 μM gliadin, (b) Absorption Spectra of aptasensors with gliadin at different

Yadi Qin, Sicheng Zhang and Jie Qian contributed equally to this work

[*] * Corresponding authors. Tel :18999250641 (J, Yao); 13999258239 (M, Zhang)

E-mail addresses: xydyaojun@163.com (J, Yao); zhang78089680@sina.com (M, Zhang)

concentrations.

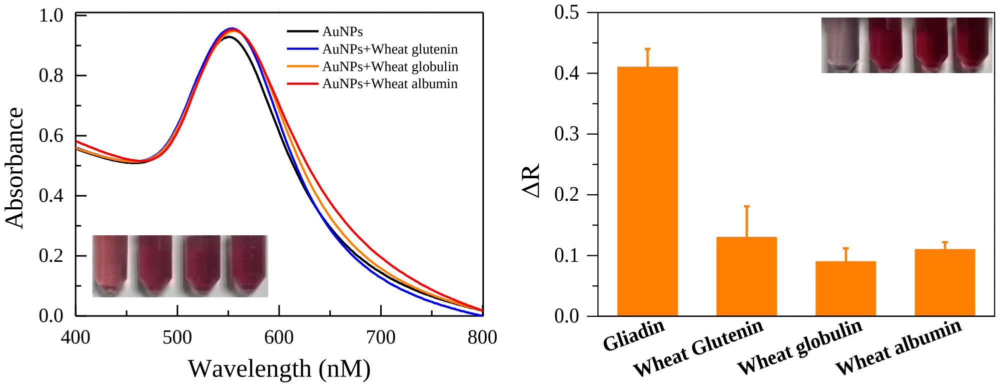

Figure S3. The selectivity of the established colorimetric sensor for analysis different proteins in flour, and the UV absorption spectra of these proteins mixed with bare AuNPs

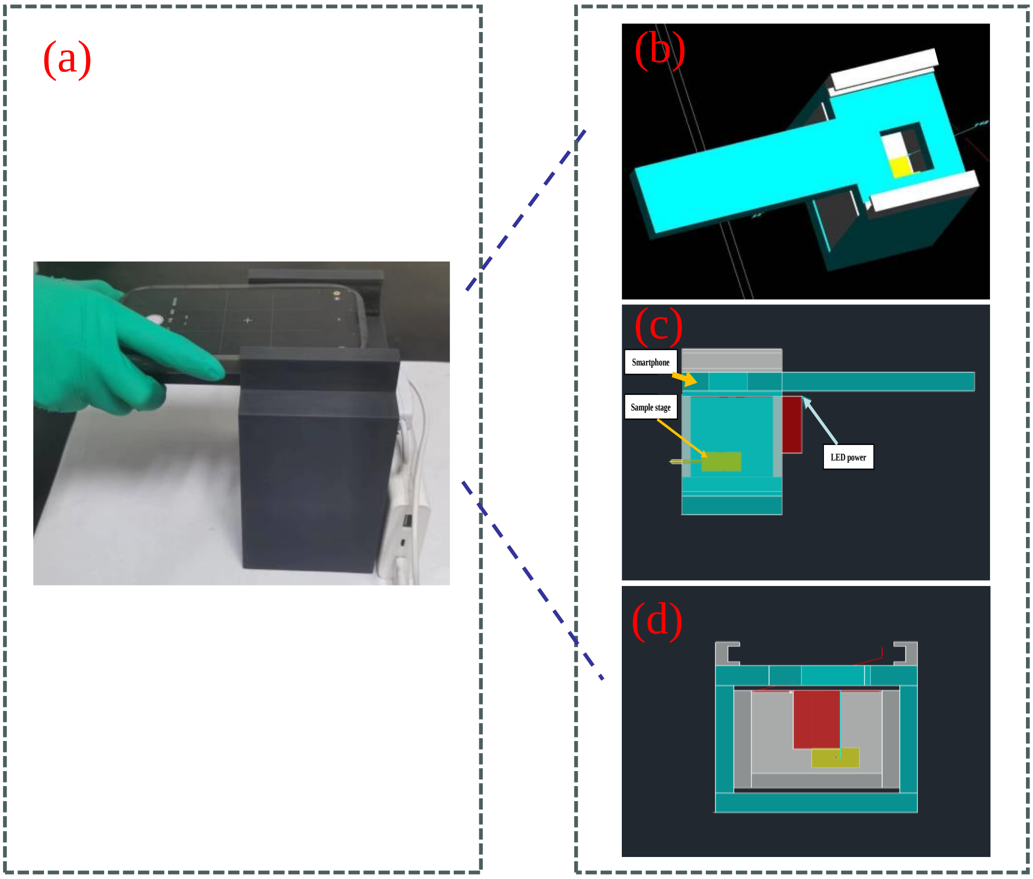

Figure S4. Physical image of portable colorimetric smartphone sensing platform(a). 3D design diagram of smartphone detection platform (b). The side of design diagram (c). The front of design

diagram (d).

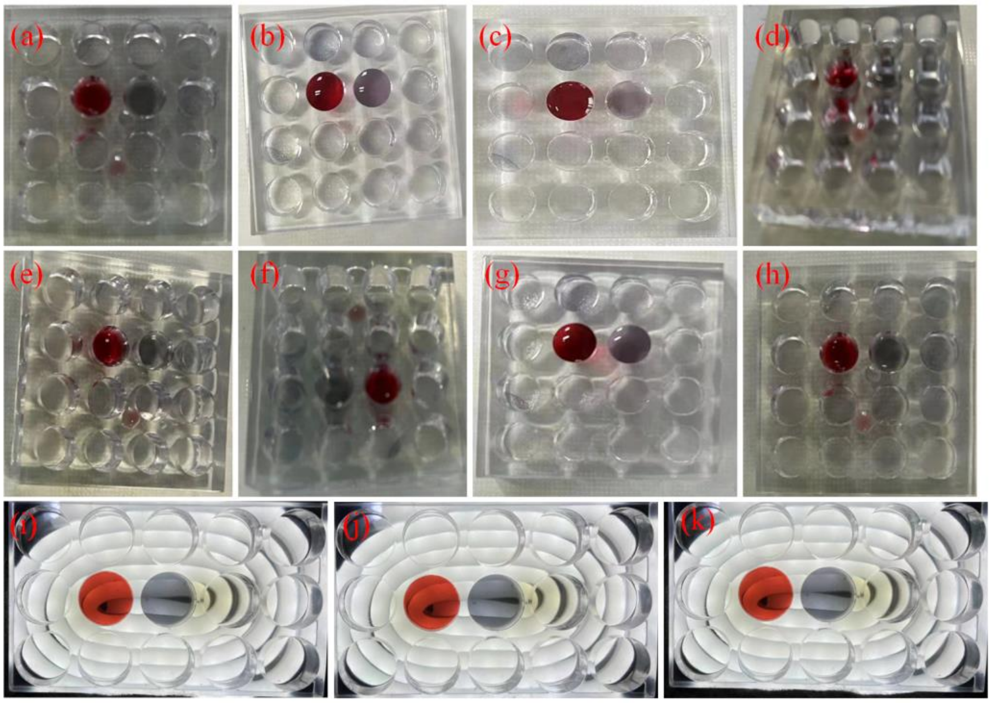

Figure S5. The color-pick images taken under different environmental conditions without using a cassette (a-h): The scanning time is at 8:00 am (a), The scanning time is at 2:00 pm (b), The scanning time is at 0:00 am (c), The camera angle is 45° (d), The camera angle is 45° (e), The focal length is 3cm(f), The focal length is 10cm (g), The focal length is 20cm (h). Using a cassette for vertical photography with a focal length of 10cm, taking color-pick images with aptasensors at different times (i-k): (i) is a image at 8:00 am, (j) is a image at 2:00 pm, (k) is a image at 0:00 am.

Table S1 The binding energy by MMPBSA (kJ/mol)

Type	Apt- Gliadins
E_{VDW}	-44.1908+/-5.9804
E_{ELE}	-122.7184+/-36.0034
E_{GB}	155.0428+/-37.9724
E_{SA}	-5.4928+/-0.9406
$G_{binding\ energy}$	-17.3592+/-5.8995

E_{VDW}: van der Waals energy
E_{ELE}: eletrostatic energy
E_{GB}: eletrostatic contribution to solvation

E_{SA}: non-polar contribution to solvation
